# Sensory optimization of crackers developed from high‐quality cassava flour, starch, and prawn powder

**DOI:** 10.1002/fsn3.431

**Published:** 2016-09-26

**Authors:** Paa T. Akonor, Nanam T. Dziedzoave, Evelyn S. Buckman, Edna Mireku Essel, Francis Lavoe, Keith I. Tomlins

**Affiliations:** ^1^ Food Research InstituteCouncil for Scientific and Industrial ResearchAccraGhana; ^2^ Food and Markets DepartmentNatural Resources InstituteUniversity of GreenwichMedway, KentUK

**Keywords:** Cassava flour, crackers, mixture design, optimization, sensory evaluation

## Abstract

Crackers produced from high‐quality cassava flour (HQCF), cassava starch, and prawn powder were optimized based on sensory preference testing. Ten different formulations of crackers were produced using mixture design. These were subjected to sensory evaluation using attributes such as taste, crispiness, puffiness, and acceptability on a 7‐point hedonic scale. A mean score of 4.7, 5.6, 5.2, and 5.2 was obtained correspondingly for taste, crispiness, puffiness, and acceptability. Scores for these attributes indicated that the crackers were acceptable. Mixture regression models were used to generate contour plots for the sensory attributes and these were superimposed to obtain an optimal region, from where an optimum formulation was chosen. Verification of the optimal formulation with acceptability studies confirmed that the newly developed snack had a likeness score of 6.3 and was highly acceptable to consumers. The study demonstrates the suitability of HQCF in processing value added snack products.

## 1 Introduction

Snack foods are popular and very well exploited throughout the world. They are handy and light and usually eaten between regular meals (Lusas, 2001). Snack foods are usually high in calories but low in protein, vitamins, and other micronutrients. Crackers made from fish or prawns, which is popular to the people of the orient, are one of the snack foods gradually making inroads into the Ghanaian eating pattern and gaining popularity. It is originally processed from tapioca starch and fish or prawn meat and utilizes the technology of puffing in its processing. The source of starch has been found to affect the quality of the final product. Starches from cassava, sago, rice, and mixtures of starches from these and other crops have been used but cassava starch is identified to result in higher expansion (Tongdang, Meenun, & Chainui, 2008).

Efforts at enhancing the potential of high‐quality cassava flour (HQCF) as an industrial raw material for food applications in Ghana continue to receive significant attention. HQCF is unfermented cassava flour produced from wholesome freshly harvested and rapidly processed cassava roots. The flour is white and odorless with low acidity and fairly neutral pH (Dziedzoave et al., 2006). Although the flour is quite versatile and applicable for use in a wide range of food products, its utilization currently, is limited to bakery and pastry products. In this study, however, HQCF was used to partially replace starch in the formulation of crackers. Whereas usage of tapioca starch in crackers has been widely reported (Cheow, Kyaw, Howell, & Dzulkifly, 2004; Kyaw, Yu, Cheow, Dzulkifly, & Howell, 2001; Saeleaw & Schleining, 2011; Tongdang et al., 2008), the application of HQCF and its combination with starch remain marginally explored. Its application in cracker production would create another avenue for profitable and diverse usage of the commodity. Additionally, it offers a cheaper alternative to processing the snack.

HQCF and cassava starch have some basic physico‐chemical and functional differences. Therefore partially replacing starch with HQCF in the formulation of crackers requires optimization. This would be useful in identifying and predicting the combinations of these ingredients that will result in a product with an ideal consumer acceptability. Sensory optimization of many food products has been reported (Bangde, Hayes, & Zeigler, 2015; Damasio, Costell, & Duran, 1999; Prinyawiwatkul, McWatters, Beuchat, & Philips, 1997). This forms an integral part of product development and facilitates the identification of sensory attributes which influence consumer acceptability (Prinyawiwatkul et al., 1997). The objective of this study, consequently, was to optimize cracker formulation from HQCF, cassava starch, and prawn powder to obtain a snack with ideal acceptability among consumers.

## 2 Methodology

Fresh black tiger prawns were obtained from a local fish market, whereas HQCF and cassava starch were procured from the Root and Tuber Products Development Unit, CSIR‐FRI. Other ingredients were purchased from a retail supermarket.

### 2.1 Processing of prawn powder

The fresh prawns were sorted, washed in potable water, peeled, and the heads removed. Afterward, the peeled prawns were spread thinly on a wire mesh and dried at 55°C for 14 hr. The dried prawn meat was milled into powder to pass through a 250 μ sieve. The prawn powder was subsequently packaged in HDPE bags, sealed, and stored in a refrigerator.

### 2.2 Formulation and production of crackers

The crackers were formulated according to a 10‐point design matrix (Table 1) from three components namely HQCF, cassava starch (CS) and prawn powder (PP). Apart from the major components, the proportion of other ingredients (salt and sugar) was kept constant in all 10 formulations.

**Table 1:** Formulae for production of the crackers

Formulation	Component		
	HQCF (%)	CS (%)	PP (%)
1	62.5	37.5	0.0
2	55.0	45.0	0.0
3	55.0	30.0	15.0
4	55.0	30.0	15.0
5	62.5	30.0	7.5
6	70.0	30.0	0.0
7	70.0	30.0	0.0
8	55.0	45.0	0.0
9	60.0	35.0	5.0
10	55.0	37.5	7.5

HQCF, high‐quality cassava flour; PP, prawn powder; CS, Cassava starch.

The HQCF, CS, and PP were mixed in a high speed laboratory blender (Waring, E8420) into uniform powder according to the formulae in Table 1. A 1:5 slurry of the powder was made with water before salt and sugar were added to taste. The slurry was cooked into a paste with continual stirring. This was done to fully gelatinize the starch and ensure full expansion during frying. The cooled paste was scooped into petri dishes and leveled before drying (60°C for 6 hr) into flat crackly pieces of about 3 mm thickness. The dried pieces were flash fried (165–175°C for less than 10 s) into dry crispy crackers in previously heated vegetable oil (*Frytol* ^*®*^) using a deep fryer (Salton Deep Fryer, SDF‐35).

### 2.3 Sensory evaluation

Thirty untrained panelists, who regularly patronize crackers and other flour‐based snacks and had previous experience in sensory evaluation, assessed the crackers in a laboratory preference test. The participants had no known allergies to any of the ingredients used. They evaluated the snack based on puffiness, crispiness, taste, and overall acceptability. A 7‐point hedonic scale (Stone & Sidel, 2004), with one representing dislike extremely and seven representing like extremely was used for the evaluation. The sensory evaluation session was conducted at an accredited facility conforming to ISO 8589 (ISO, 2007) and equipped with individual sensory booths. Samples were presented to panelists following a randomized design matrix (XLSTAT ver. 2014, Statsoft, France). Panelists were instructed to refresh their palate with a slice of fresh cucumber and rinse with noncarbonated water before tasting subsequent samples.

### 2.4 Experimental design and data analysis

Based on findings from preliminary studies, a 3‐component mixture experiment (simplex centroid) was designed using the following lower and upper constraints: 55%–70% (HQCF), 30%–45% (CS), and 0%–15% (PP) (Minitab 17.0.1). This resulted in a 10‐point design matrix (Table 1). Results from the study were analyzed using mixture regression, to fit the data to Scheffe polynomial models (Minitab 17.1.0) of the form *Y * =  β_*1*_ *X*_*1*_+ β_*2*_ *X*_*2*_+ β_*3*_ *X*_*3*_+ β_*12*_ *X*_*1*_ *X*_*2*_+ β_*13*_ *X*_*1*_ *X*_*3*_+ β_*23*_ *X*_*2*_ *X*_*3*_ where *Y* is the predicted dependent variable (taste, puffiness, crispiness, and overall acceptance) and β_*i*_ is parameter estimates for linear and cross‐product terms; *X*_*1*_ **= HQCF, *X*_*2*_ **= CS, and *X*_*3*_ = PP. These polynomial models were then used to generate contour plots for the dependent variables.

### 2.5 Optimization and validation of optimum formulation

Contour plots for the various attributes (taste, crispiness, puffiness, and acceptability) were superimposed to obtain an optimal region. The ranges were 5.5–6.5 for taste, 6.0–6.5 for crispiness, and puffiness and 6.0–7.0 for overall likeness. Crackers made from a formulation selected from this region were subjected to a consumer acceptance test. The scores from the consumer acceptance test were used to validate the regression models.

### 2.6 Consumer acceptance test

A consumer acceptance test was done to validate the optimal formulation. Seventy‐five consumers were recruited to participate in a consumer acceptance survey. The selected participants had no known food allergies and were consumers (both casual and regular) of snack products, including crackers. Recruitment was based on interest and availability, after which panelists were made to sign a consent form. Based on results from the sensory evaluation, the optimal formulation was scaled up and used in this survey. Each participant was served with 5 g of deep‐fried HQCF crackers (prepared as described in section 2.3) on a white Styrofoam disposable plate labeled with a 3‐digit blinding code. Participants were made to taste and rate their degree of liking of the snack on a 7‐point hedonic scale with 1‐dislike extremely and 7‐like extremely, based on attributes such as taste, crispiness, puffiness, and overall acceptability.

### 2.7 Statistical analysis

Individual scores from the physical and sensory evaluation were averaged and data analyzed (SPSS 17.0.1). Significantly different means were separated using Duncan's Multiple Range Test (DMRT) at 95% confidence interval. Data from the consumer acceptance survey were also coded and analyzed using SPSS.

## 3 Results and Discussions

### 3.1 Sensory evaluation

Table 2 presents the mean ratings for the attributes assessed in the laboratory preference test. The results show variations in the 10 formulations as far as these attributes are concerned. Some of the compositions yielded quite similar scores and may aggregate into common homogenous groups (an indication that likeness for these formulations are similar).

**Table 2:** Mean score for preference of sensory attributes

Formulation	Mean scores for sensory attributes^a^			
	Taste	Crispiness	Puffiness	Acceptability
1	3.37 ± 1.74	4.63 ± 1.54	4.89 ± 1.41	4.91 ± 1.71
2	2.95 ± 1.85	4.35 ± 1.79	5.05 ± 1.35	4.40 ± 1.79
3	5.30 ± 1.42	5.30 ± 1.45	4.23 ± 1.48	5.42 ± 1.54
4	5.40 ± 1.33	5.25 ± 1.28	3.15 ± 1.63	5.40 ± 1.50
5	5.10 ± 1.71	6.10 ± 1.83	5.30 ± 1.34	5.53 ± 1.65
6	5.30 ± 1.84	6.45 ± 1.28	6.35 ± 1.31	4.70 ± 1.64
7	5.40 ± 1.26	6.48 ± 1.05	6.40 ± 1.28	5.60 ± 1.07
8	4.01 ± 1.06	5.33 ± 1.11	5.10 ± 1.46	4.52 ± 1.33
9	4.45 ± 1.54	5.90 ± 1.68	5.10 ± 1.33	5.10 ± 1.67
10	6.50 ± 0.95	6.15 ± 1.42	6.15 ± 1.04	6.60 ± 0.97

a Sensory attributes were rated on a scale of 1 (dislike extremely) – 7 (like extremely).

Taste is a key component in sensory evaluation and an important primary attribute that influences the acceptability of almost every food product. As shown (Table 2), the scores for taste ranged between 3.0 and 6.5, with a mean of 4.77, suggesting that the taste of the newly developed snack was generally acceptable to the panelists. Indeed, most of the formulations received a score of 5 or more, which represents, at least, “like moderately” on the 7‐point hedonic scale.

**Figure 1:**
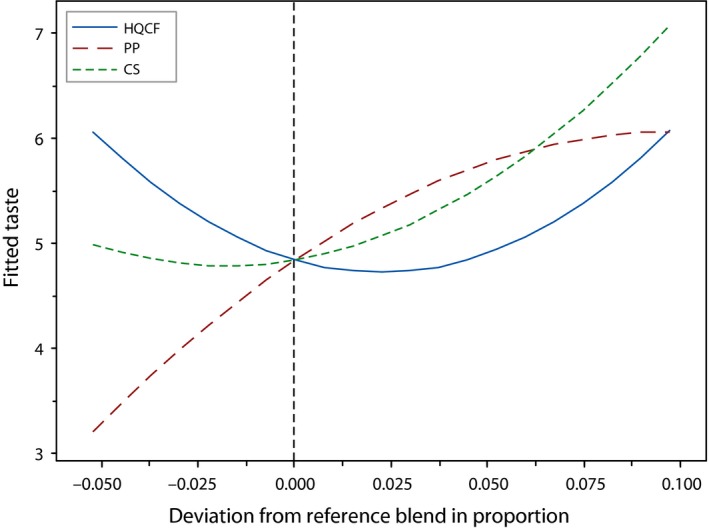
Cox response trace plot for taste of crackers, where HQCF‐high‐quality cassava flour, PP‐prawn powder, CS‐Cassava starch

The effect of changes in proportion of each component on taste of the crackers is illustrated in the cox response trace plot (Figure 1). The trace plot shows that an all three components affected the taste of the product. Increasing proportions of both CS and PP caused a rapid increase in taste rating of the prawn crackers. Binary combinations of these two ingredients significantly (*p* < .05) influenced the taste of the product. Generally, formulations with 0% PP were rated lower compared to higher and intermediary amounts of the same ingredient. Also, an increase in CS resulted in an improvement in taste, as perceived by the panelists. A previous study by Irianto and Sugiyono & Indriati (2002) showed that intermediary amounts of tapioca flour and fish were optimal for obtaining high taste appeal for fish crackers. The mixture regression showed that taste was influenced by only the quadratic term in the model. This model was reliable (*R* ^2^ = 92.2%) in predicting the taste of the newly developed product, with a nonsignificant lack of fit (*p* = .340).

Texture of deep‐fried snacks is one of its essential attributes, with crispy and crackly ones being the most preferred. As indicated by Saeleaw and Schleining (2011), brittleness with sound emission at a low fracture force are characteristic of crispiness. Similar to most fried snacks, crispiness is an important indicator of the quality of crackers. The ratings for this attribute ranged between 4.4 and 6.5, with significant differences (*p* < .05) existing between different formulations. Generally, binary blends of HQCF and CS containing high amounts of the former, and low amounts of the latter returned higher scores for crispiness. On the contrary, PF negatively affected crispiness of the crackers. This observation, according to Yu (1991), may be attributed to the fact that addition of PF reduces the total amylopectin fraction of the flour/starch.

**Figure 2:**
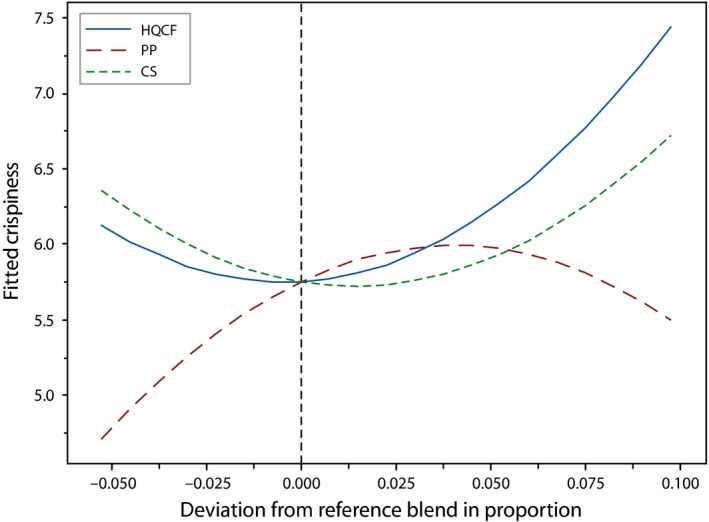
Cox response trace plot for crispiness of crackers, where HQCF, high‐quality cassava flour; PP, prawn powder; CS, Cassava starch

Crispiness correlates well with expansion (Neiva et al., 2011; Kyaw et al., 2001) and is also associated with the total amylopectin content of the flour or starch used. Yu (1991) also suggested that fish protein hinders expansion through its interaction with starch granules, and therefore high fish protein ultimately reduced the crispiness of fish crackers. This observation is consistent with Irianto and Sugiyono & Indriati (2002), who also obtained reduced crispiness in fish crackers at higher fish:starch ratios. Figure 2 indicates that HQCF had a stronger influence on the crispiness compared to CS and also reveals the negative effect of increasing amounts of PP on crispiness. The predictive model for crispiness was reasonably accurate, explaining nearly 90% of the variability in the crispiness rating of the crackers (adj *R* ^2^ = 76.4%), with a nonsignificant lack of fit (Table 3).

**Table 3:** Regression models for sensory attributes

Predictor variables^a^	Coefficient estimates (amounts)			
	Taste	Crispiness	Puffiness	Acceptability
HQCF	0.3890	0.3242	0.3251	0.1005
CS	0.8160	0.6367	0.6739	0.1182
PP	−0.8464	−1.1797	−1.3547	−0.7034
HQCF×CS	−0.0220^a^	−0.0168	−0.0174	−0.0026
HQCF×PP	−0.0079	0.0054	0.0021	−0.0004
CS×PP	0.0336^a^	0.0207^a^	0.0288^a^	0.0254^a^
R^2^	92.17	89.49	91.84	78.87
Adj. R^2^	82.37	76.36	81.64	52.46

HQCF, high‐quality cassava flour; PP, prawn powder; CS, Cassava starch.

a denotes significant coefficient at *p* < .05.

**Figure 3:**
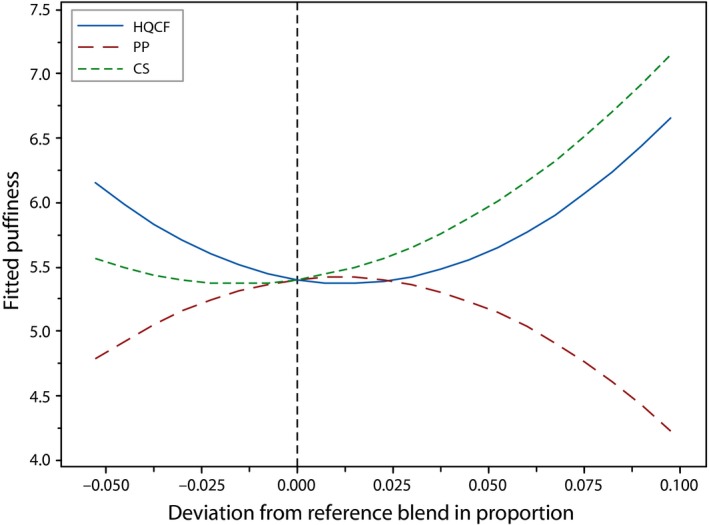
Cox response trace plot for puffiness of crackers, where HQCF, high‐quality cassava flour; PP, prawn powder; CS, Cassava starch

Expansion is critical in deciding the final quality of crackers and largely correlates strongly with acceptability (Nurul, Boni, & Noryati, 2009; Taewee, 2011). Puffing has been described by Rakesh and Datta (2011) as a rapid heating phenomenon in food which changes its structure. In crackers, it occurs when moisture trapped within starch granular matrix instantaneously evaporates because of exposure to high oil temperatures. Puffiness scores ranged from 3.2 to 6.4. Formulations 3 and 4, which were ternary blends and incidentally contained the highest proportion of PF, had the lowest puffiness appeal. Conversely, formulations 6 and 7, which were binary blends of HQCF and CS, obtained the highest rating for this attribute. As shown in Figure 3, increasing the proportion of HQCF and CS increased puffiness rating, whereas an increase in PP resulted in the opposite. This observation corroborate previous studies by Cheow, Yu, Howell, Che Man, and Muhammad (1999), Huda et al. (2001) and Kyaw et al. (2001) who reported a negative correlation between the proportion of fish and linear expansion of fish crackers.

Linear expansion, and hence puffiness, is widely known to be affected by fish/starch ratio. Although the mechanism seem complex and not well elucidated, Kyaw et al. (2001) explained that high protein may obstruct gelatinization of starch. Puffiness was significantly influenced by the quadratic terms in the model as well as the cross product of CS and PP (Table 3). The model for puffiness was significant (*p* = .027) and convincingly accurate (*R* ^2^ = 91.8%) in fitting the experimental data for this attribute.

The crackers were largely acceptable to the panelist. A mean score of 5.2, representing ‘like moderately’, was obtained for overall acceptability of the crackers (Table 2). Only Formulation two fell below the threshold (5) for likeness, on the 7‐point hedonic scale. Notwithstanding this narrow variation, significant differences (*p* < .05) were noticed in the acceptability scoring of crackers from the various runs. Whereas ternary blends attained a score of 5 or more, a trend of reduced acceptability rating was observed for binary combinations of HQCF and CS. Incidentally formulation 10, a ternary blend containing moderate proportion of the ingredients, had the highest acceptability rating.

**Figure 4:**
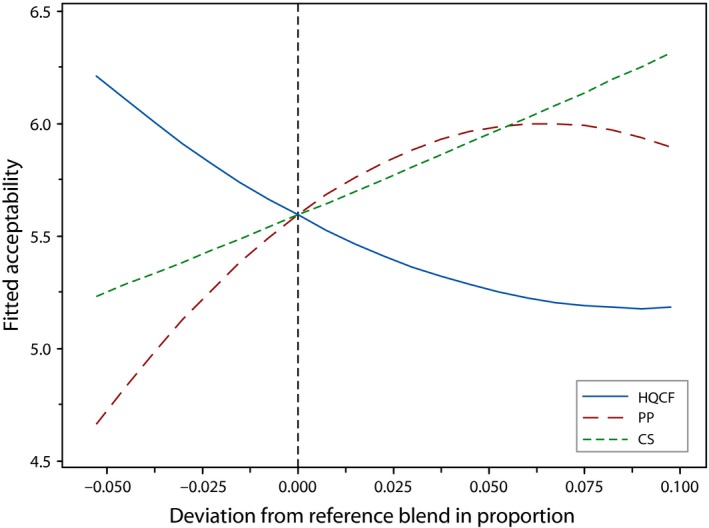
Cox response trace plot for acceptability of crackers, where HQCF, high‐quality cassava flour; PP, prawn powder; CS, Cassava starch

The cox response trace plot in Figure 4 illustrates the effect of changes in proportion of the ingredients on the overall liking of prawn crackers. While acceptability of crackers increased as HQCF approached its lower bound, an increase in the proportions of each of CS and PP rapidly enhanced the acceptability rating. The regression model explained almost 80% (adj. *R* ^2^ = 52.5%) of the variation in overall acceptance with no lack‐of‐fit (*p* = .191). Apart from CS*PP, none of the terms in the model were significant.

### 3.2 Optimal Formulation

**Figure 5:**
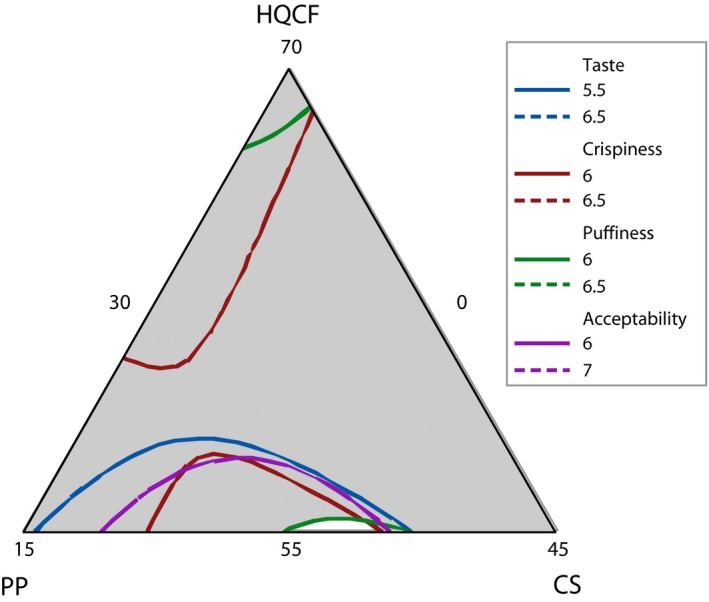
Overlaid contour plots showing optimal region (unshaded area), where HQCF, high‐quality cassava flour; PP, prawn powder; CS, Cassava starch

Contour plots of the individual attributes were superimposed to obtain an optimal region (unshaded area in Figure 5) where specified conditions of the sensory attributes were satisfied. A formulation from within this region, containing 55.2% HQCF, 5.3%PP, and 39.5% CS was chosen to verify the predicted models. Mean rating for consumer assessment test is presented in Table 4. The results indicate that the ratings from the consumer test compared very well with the predicted values. Generally, a deviation of less than 1 was observed between the predicted and observed score, an observation which authenticates the regression models.

**Table 4:** Predicted and observed sensory ratings of optimal formulation

Sensory attribute	Predicted score	Observed score	Deviation
Taste	5.8	6.4	0.4
Crispiness	6.0	5.9	0.1
Puffiness	6.0	5.7	0.2
Acceptability	6.1	6.3	0.1

## 4 Conclusion

The study has shown that the crackers produced from HQCF, prawn powder, and starch were generally acceptable to the consumers, although perceptible differences were noticed in the sensory properties of some combinations. The panelists preferred formulations composed of fairly lower proportion of HQCF and intermediary amounts of prawn powder and starch. Prawn powder and starch greatly influenced most of the sensory attributes of the snack. The formulation composed of 55% HQCF, 5% PP, and 40% CS, obtained from the optimal region was highly acceptable by consumers, with an overall likeness score of 6.3 (like very much).

## Conflict of Interest

None declared.
